# Active Switching Between Absorption and Polarization Conversion Enabled with VO_2_-Based Reconfigurable Terahertz Metasurfaces

**DOI:** 10.3390/nano16171054

**Published:** 2026-08-24

**Authors:** Danyan Lu, Yizhen Lin, Junjie Song, Jiarui Li, Yue Zhou, Mingzhong Wu, Wei Wang, Xunjun He

**Affiliations:** 1 Department of Electronic Science and Technology, School of Electrical and Electronic Engineering, Harbin University of Science and Technology, Harbin 150080, China; 2 Harbin Electric Machinery Company Limited, Harbin 150040, China

**Keywords:** reconfigurable metasurface, VO_2_, absorption, PC, function switching

## Abstract

Terahertz (THz) metasurfaces have drawn considerable research interest, owing to their compelling potential in sensing, imaging, and wireless communication. However, most existing designs are constrained to a single predefined function, severely hindering their practical applicability in dynamic or multifunctional scenarios. Herein, we present a reconfigurable THz metasurface that enables on-demand functional transformation by harnessing the phase transition characteristics of vanadium dioxide (VO_2_). The designed unit cell adopts a six-layer stacked configuration, sequentially comprising a VO_2_ square ring, a first polyimide (PI) dielectric spacer, an elliptical gold patch, an intermediate VO_2_ thin film, a second PI dielectric spacer, and a gold ground plane. When VO_2_ is in its metallic phase, the metasurface operates as a metal–insulator–metal (MIM) absorber, achieving over 90% absorption in the frequency range of 1.01–1.91 THz. In the insulating state, it acts as a polarization converter, enabling efficient linear-to-circular polarization conversion (PC) with an axial ratio (AR) below 3 dB from 1.82 to 2.21 THz under linearly polarized (LP) incidence. Moreover, the metasurface exhibits robust performance under varying incident angles and different polarization conditions. Collectively, this design offers a flexible and reconfigurable platform for advanced THz devices, laying a solid foundation for THz communication, intelligent sensing, and imaging systems.

## 1. Introduction

The rapid progress of THz technology has fueled growing demand for functional electromagnetic devices in diverse fields, including nondestructive evaluation [1,2,3], spectroscopy [4,5,6], imaging [7,8,9], and high-speed wireless communications [10,11]. Among these promising candidates, metasurfaces, artificial arrays of subwavelength elements, have emerged as powerful platforms for precisely manipulating the phase, amplitude, and polarization of electromagnetic waves [12]. However, traditional metasurfaces are predominantly passive, with a fixed response upon fabrication, rendering them incapable of adapting to dynamic or real-time operational conditions, as similarly observed in conventional MIM absorbers [13]. Consequently, this inherent limitation has created a pressing demand for tunable or reconfigurable metasurfaces capable of actively tailoring their properties in response to external stimuli.

To enable tunable or reconfigurable metasurfaces, various active materials, including graphene [14,15,16], semiconductors [17], liquid crystals [18], and Ge_2_Sb_2_Te_5_ [19], have been widely integrated into THz metasurface architectures. For instance, Song et al. achieved switching between narrowband and broadband absorption via VO_2_ phase transition [20]. Cheng et al. demonstrated a dual-function THz absorber leveraging the electrically tunable optical response of graphene [21]. Zhang et al. actively tuned absorption frequency and amplitude by varying the Fermi level and relaxation time of graphene [22]. Additionally, graphene-assisted designs have also been extended to biosensing and polarization-dependent applications [23,24]. Hou et al. realized a linear-to-circular PC (LCPC) with dynamically controllable ellipticity [25]. Collectively, these studies confirm that active materials significantly enhance the tunability and adaptability of THz metasurfaces compared with conventional passive counterparts. Despite these advances, most existing works remain focused on continuous tuning of a single function, such as absorption enhancement, bandwidth broadening, or polarization manipulation.

Compared with other phase-change materials (PCMs) such as Ge_2_Sb_2_Te_5_ (GST), Sb_2_S_3_, and GeSe [19], VO_2_ offers several compelling advantages: an abrupt and reversible insulator-to-metal transition near 68 °C with multidecade conductivity modulation, a relatively low transition temperature for practical thermal actuation, proven compatibility with THz metasurface fabrication, and volatile reconfiguration that eliminates the need for reset pulses. Exploiting these merits, numerous VO_2_-integrated metasurfaces have been developed for various switchable functionalities, including absorption-to-focusing [26], absorption-to-asymmetric transmission (AT) [27], absorption-to-PC [28,29,30,31,32,33,34], and tri-mode switching among absorption, transmission, and reflection [35]. Among various reconfigurable functionalities [36,37,38,39,40], the switching between absorption and PC is particularly appealing for practical THz systems. In many application scenarios, such as dynamic radar cross-section management, secure wireless links, sensing, and adaptive imaging, the system requires either perfect absorption to suppress unwanted reflections or controlled polarization manipulation to enhance signal integrity or channel isolation. Despite significant progress, most existing designs typically suffer from limited bandwidth, low angular stability, and polarization-sensitive responses, which severely hinder their practical application. Hence, there remains a pressing need for compact multifunctional platforms that can dynamically toggle between distinct electromagnetic responses within a single architecture.

To overcome these limitations, we propose a VO_2_-based reconfigurable THz metasurface that enables functional switching within a compact single structure. The unit cell comprises a six-layer stack: a VO_2_ ring, a first PI dielectric spacer, an elliptical gold patch, an intermediate VO_2_ film, a second PI spacer, and a gold ground plane. By leveraging the VO_2_ phase transition, the metasurface achieves high-efficiency absorption in the metallic state and high-quality LCPC in the insulating state. Beyond the mere integration of two functions, the proposed design introduces several distinctive advances at both conceptual and performance levels. First, unlike most prior VO_2_-based switchable designs that operate in narrow bands or at lower frequencies, our structure simultaneously delivers a broad absorption bandwidth of 0.90 THz (1.01–1.91 THz) with over 90% average absorption and an LCPC band of 0.39 THz (1.82–2.21 THz) at higher frequencies within a passive, thermally switchable platform. Second, the absorption mode exhibits full polarization insensitivity and an angular stability up to 55°, which is rarely achieved in previously reported switchable metasurfaces and is critical for practical applications where incident polarization and angle cannot be controlled. Third, the six-layer architecture, though seemingly complex, provides independent design freedom for optimizing each mode without compromising the other, offering a systematic route toward multifunctional integration beyond simple stacking of existing designs. Fourth, the proposed configuration achieves these performances without relying on complex biasing networks or active electronic controls, instead relying solely on the intrinsic phase transition of VO_2_, which simplifies fabrication and operation. Collectively, these features, including broadband and high-frequency operation, polarization insensitivity, superior angular robustness, and structural versatility, establish the proposed metasurface as a conceptually new platform for reconfigurable THz devices, offering a clear advance over existing switchable designs and providing a practical pathway toward compact, adaptive THz systems for wireless communication, intelligent sensing, and imaging applications.

## 2. Designs and Simulations of Unit Cells

**Figure 1:**
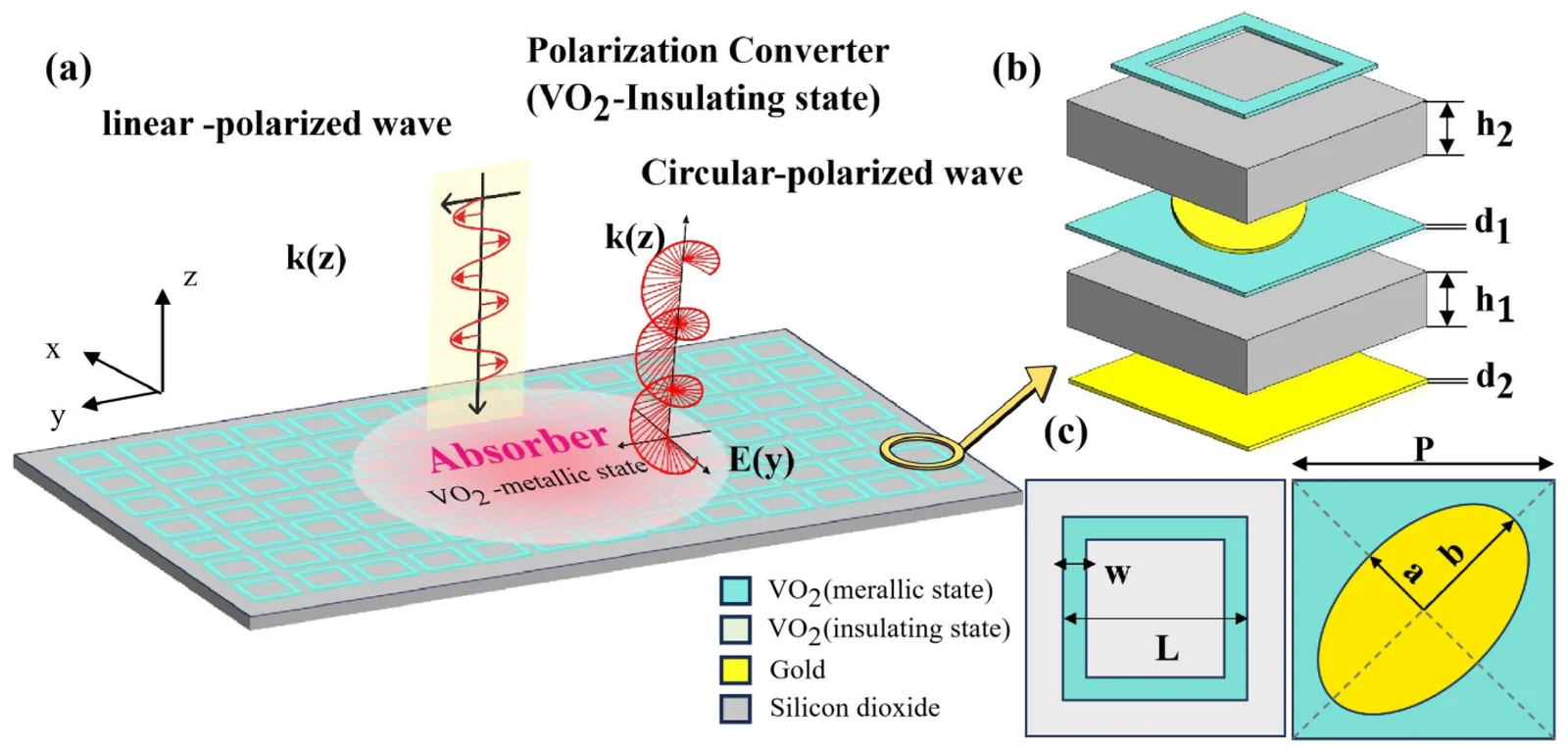
Schematic diagram of a VO_2_-based reconfigurable THz metasurface: (**a**) functional switching under different states of VO_2_, (**b**) three-dimensional structure of the unit cell, and (**c**) structures of the top ring and elliptical gold patch.

To achieve active switching between absorption and PC, we design a VO_2_-based reconfigurable THz metasurface, as illustrated in Figure 1. The unit cell adopts a six-layer stacked configuration (Figure 1b): a top VO_2_ ring, a first PI spacer, an elliptical gold patch, an intermediate VO_2_ layer, a second PI spacer, and a gold ground plane. Although the structure comprises six layers, each layer plays a distinct and indispensable role in the dual-function operation. When VO_2_ is in the metallic state, the top VO_2_ ring, the first PI spacer, and the intermediate VO_2_ film form a complete MIM cavity that supports Fabry-Pérot (F-P) resonance for broadband absorption [30,41,42]. In the insulating state, since the intermediate VO_2_ becomes transparent, the elliptical gold patch, the second polyimide spacer, and the bottom gold plane act as an anisotropic reflective polarizer, enabling LCPC. The two dielectric spacers are necessary because the absorption and PC mechanisms rely on different resonant cavities with distinct thickness requirements. The intermediate VO_2_ layer functions as a switchable mirror for absorption and a passive spacer for PC, while the bottom gold plane serves as a common reflector for both modes. Even in reflection mode, a back reflector is necessary to realize near-unity reflection and the phase control required for PC. Removing any layer would inevitably disable or severely degrade one of the two functions. Thus, this multilayer architecture provides independent design freedom for optimizing both responses within a single platform. Once heated, VO_2_ undergoes a temperature-induced monoclinic-to-tetragonal phase transition near 68°, enabling reversible switching between insulating and metallic states [26,27]. In the metallic state, the MIM-based F-P cavity confines incident energy via magnetic resonance [43,44] and dissipates it through ohmic and dielectric losses, yielding high-efficiency absorption with excellent angular insensitivity owing to the structural symmetry of the top ring (Figure 1a). In the insulating state, the elliptical patch introduces geometric anisotropy, producing distinct reflection amplitudes and phase responses for orthogonal polarizations. With optimized dimensions, equal amplitudes and a 90° phase difference are achieved, enabling LCPC in reflection mode (Figure 1a). Consequently, the proposed metasurface offers actively switchable absorption and PC within a compact platform.

To validate the above-described performances, full-wave finite-difference time-domain (FDTD) simulations are performed with periodic boundaries along the *x*- and *y*-directions and open boundaries along the *z*-direction. The optimized geometric parameters are: P = 50 μm, $h_{2}\;=\;27\;\mu m$, $h_{1}\;=\;12\;\mu m$, $d_{1}\;=\;0.2\;\mu m$, $d_{2}\;=\;0.5\;\mu m$, L = 38.5 μm, w = 3.5  μm, a = 17 μm, b = 22 μm. The PI spacer has a relative permittivity *ε*ᵣ = 3.5 and loss tangent tg *δ* = 0.0027. The effective permittivity of VO_2_ is modeled using the effective medium theory (EMT) [31,45,46]: $\varepsilon_{c}\;=\;\frac{1}{4}\left[\varepsilon_{D}\left(2\;-\;3f\left(T\right))\;+\;\varepsilon_{M}(3f\left(T\right)\;-\;1\right)\;+\;\sqrt{{\left(\varepsilon_{D}\left(2\;-\;3f\left(T\right)\right)\;+{\;\varepsilon }_{M}\left(3f\left(T\right)\;-\;1\right)\right)}^{2}\;+\;8\varepsilon_{D}\varepsilon_{M}}\right]$, where f (T) is the metallic-phase volume fraction. The insulating state is described by $\varepsilon_{D}\;=\;9$, while the metallic state follows the Drude model: $\varepsilon_{M\;=\;}\varepsilon_{\infty }\;-\;\frac{\omega_{p}^{2}\left({\mathrm{VO}}_{2}\right)}{\omega^{2}\;+\;i\gamma \omega }$, with $\varepsilon_{\infty }$ = 12 as the high-frequency dielectric constant and γ = 5.75 × $10^{13}$ rad/s as the collision frequency, and ω as the angular frequency of the incident wave. The plasma frequency $\omega_{p}^{2}\left({\mathrm{VO}}_{2}\right)$ depends on conductivity σ via $\omega_{p}^{2}\left({\mathrm{VO}}_{2}\right)\;=\;\frac{\sigma }{\sigma_{0}}\omega_{p}^{2}\left(\sigma_{0}\right)$, where $\sigma_{0}$ = 3 × 10^5^ S/m is the reference conductivity and $\omega_{p}(\sigma_{0}$) = 1.4 × $10^{15}$ rad/s is the corresponding plasma frequency. Moreover, the Drude-EMT framework has been extensively validated by terahertz time-domain spectroscopy (THz-TDS) experiments on VO_2_ thin films [47]. Additionally, VO_2_ undergoes a reversible insulator-to-metal transition (IMT) at approximately 68 °C (341 K), accompanied by a conductivity increase in four to five orders of magnitude [48]. Accordingly, we set $\sigma_{{\mathrm{VO}}_{2}}$ to 200 S/m for the insulating state and 2 × 10^5^ S/m for the metallic state, consistent with reported experimental values [49,50].

## 3. Results and Discussion

Owing to the reversible phase transition of VO_2_, the proposed metasurface can be reconfigured between two distinct functionalities: a broadband absorber in the metallic state and a polarization converter in the insulating state. The performance of each mode is detailed in the following subsections.

### 3.1. Absorber

For metasurface-based absorbers [51], the absorption is defined as $A=1-R-T=1-|S_{11}{|}^{2}-|S_{21}{|}^{2}$, where R and T are reflection and transmission intensities, respectively. In our simulations, the specific conductivity σ = 187,013 S/m corresponds to the near-fully metallic state of VO_2_, where the top ring possesses sufficient conductivity to sustain the MIM cavity resonance and realize impedance matching to free space. The intermediate value σ = 20,000 S/m represents a partial metallic state during the phase transition, illustrating the gradual evolution of absorption.

**Figure 2:**
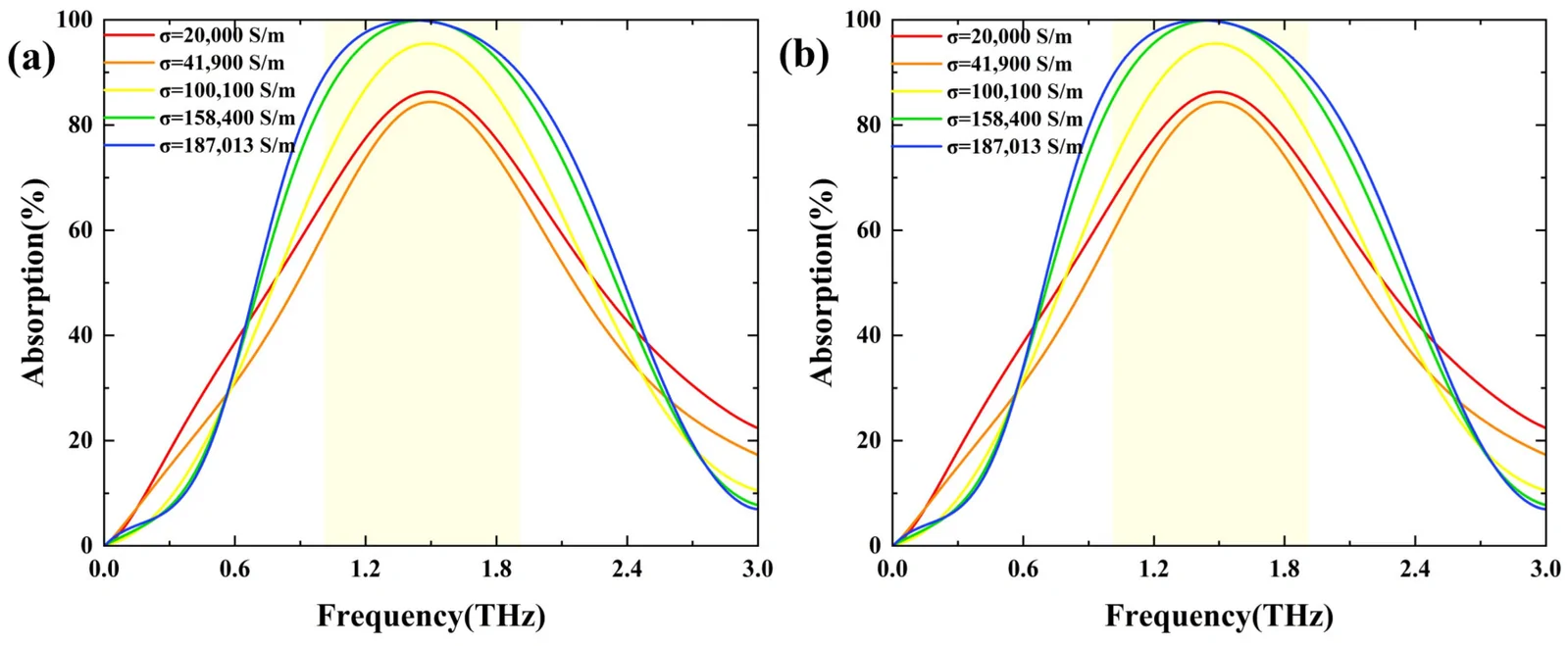
Absorption intensity of the metasurface with different VO_2_ conductivities under different incidences: (**a**) TE and (**b**) TM.

Figure 2a shows the absorption spectra under normal TE incidence for various conductivities. At *σ* = 20,000 S/m, the partial metallic top ring causes severe impedance mismatch and low ohmic loss, resulting in weak absorption across the 0–3 THz. As the phase transition proceeds, the top ring becomes increasingly metallic and *σ* increases [40]. At σ  = 187,013 S/m, impedance matching is achieved, yielding over 90% absorption from 1.01 to 1.91 THz and exceeding 80% absorption over a broader band of 0.90–2.11 THz. Compared with previously reported values [51], our absorption bandwidth enables significant enhancement. Under TM incidence (Figure 2b), the absorption evolution is nearly identical, further confirming structural isotropy. This polarization insensitivity is inherently guaranteed by the fourfold rotational symmetry of the top square-ring geometry, that is, the structure is invariant under 90° rotations, so the excitation of the MIM cavity resonance and the impedance matching to free space are independent of the in-plane polarization direction. Accordingly, the TE and TM absorption spectra are virtually identical across the entire frequency range, consistent with the symmetry analysis presented later in Figure 5. Thus, in its metallic phase, this metasurface acts as a high-efficiency, polarization-insensitive absorber.

**Figure 3:**
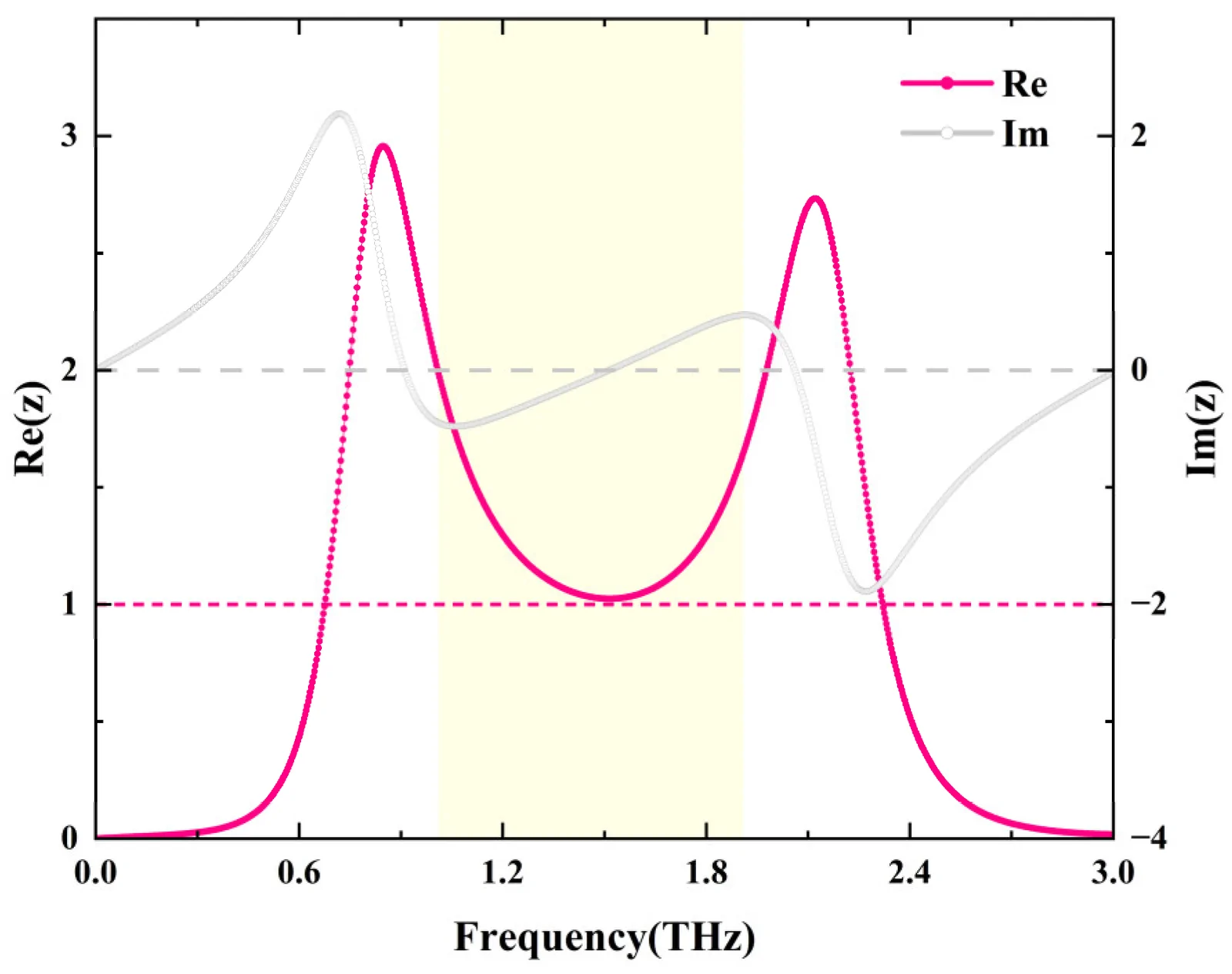
Re(z) and Im(z) of the normalized input impedance in the metallic state.

**Figure 4:**
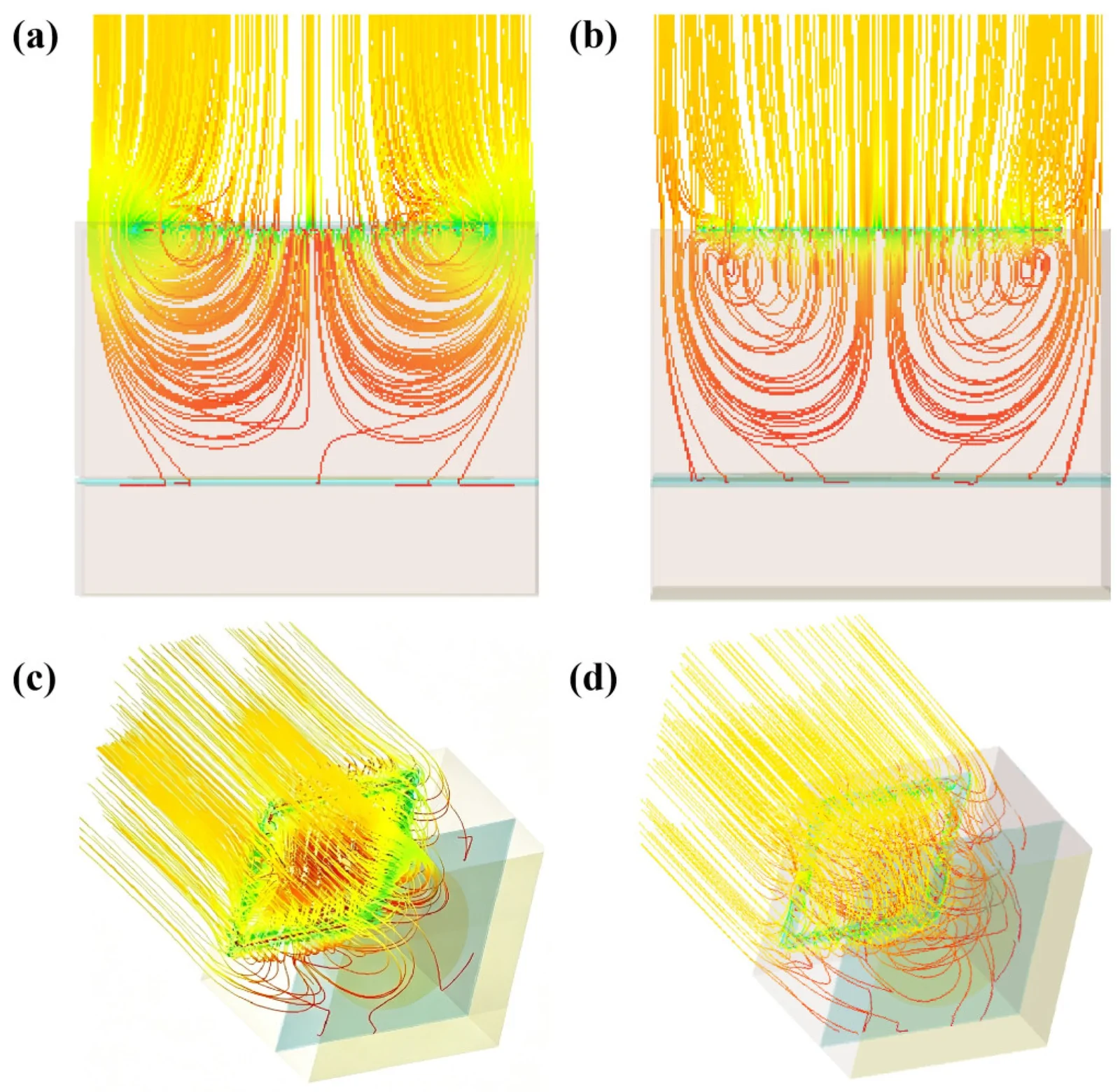
Simulated power flow streamlines within the unit cell at the absorption mode: (**a**,**b**) XOZ-plane views separately at 1.0 THz and 1.7 THz; (**c**,**d**) 3D flux lines separately at 1.0 THz and 1.7 THz.

To elucidate the absorption mechanism, we first extract the normalized input impedance using $z(\omega )\;=\;Z(\omega )/Z(0)\;=\;(1\;+\;S_{11})/(1\;-\;S_{11})$ [52,53]. Perfect absorption requires impedance matching to free space, i.e., z(ω) = 1, which corresponds to Re(*z*) = 1 and Im(*z*) = 0. As shown in Figure 3, at σ = 187,013 S/m, Re(z) remains close to unity and Im(z) stays near zero across the 1.01–1.91 THz band, confirming resonant coupling and efficient energy dissipation. To gain deeper insight, we analyze the power flow distributions within the metasurface at 1.0 THz and 1.7 THz, which directly visualize the energy transport and dissipation pathways, as shown in Figure 4. At 1.0 THz (Figure 4a,c), the streamlines exhibit pronounced meandering and swirling trajectories around the top VO_2_ ring, indicating a strong localized magnetic resonance at the ring edges. The streamlines then propagate downward along the ring edges through the PI spacer and are abruptly truncated at the bottom VO_2_ layer, confirming complete absorption without transmission. This behavior is characteristic of a magnetic resonance, where antiparallel surface currents between the top ring and bottom VO_2_ layer generate a magnetic dipole moment, and the energy is dissipated primarily through ohmic losses in the metallic ring and dielectric losses in the underlying PI spacer [54]. In contrast, at 1.7 THz (Figure 4b,d), most streamlines penetrate straight through the center region of the top ring and converge within the PI spacer without reaching the bottom layer, indicating that absorption is dominated by Fabry-Pérot cavity resonance, where energy is confined and dissipated through multiple internal reflections and dielectric losses [55]. The distinct spatial distributions reveal a frequency-dependent shift from the ring-edge region at lower frequencies to the cavity center at higher frequencies, consistent with the dispersive nature of the MIM cavity [56]. Collectively, the power flow analysis confirms that the absorber efficiently traps and dissipates incident THz energy via a combination of magnetic resonance and Fabry-Pérot cavity effects. Together with the impedance matching analysis, this provides a comprehensive and intuitive understanding of the absorption behavior.

**Figure 5:**
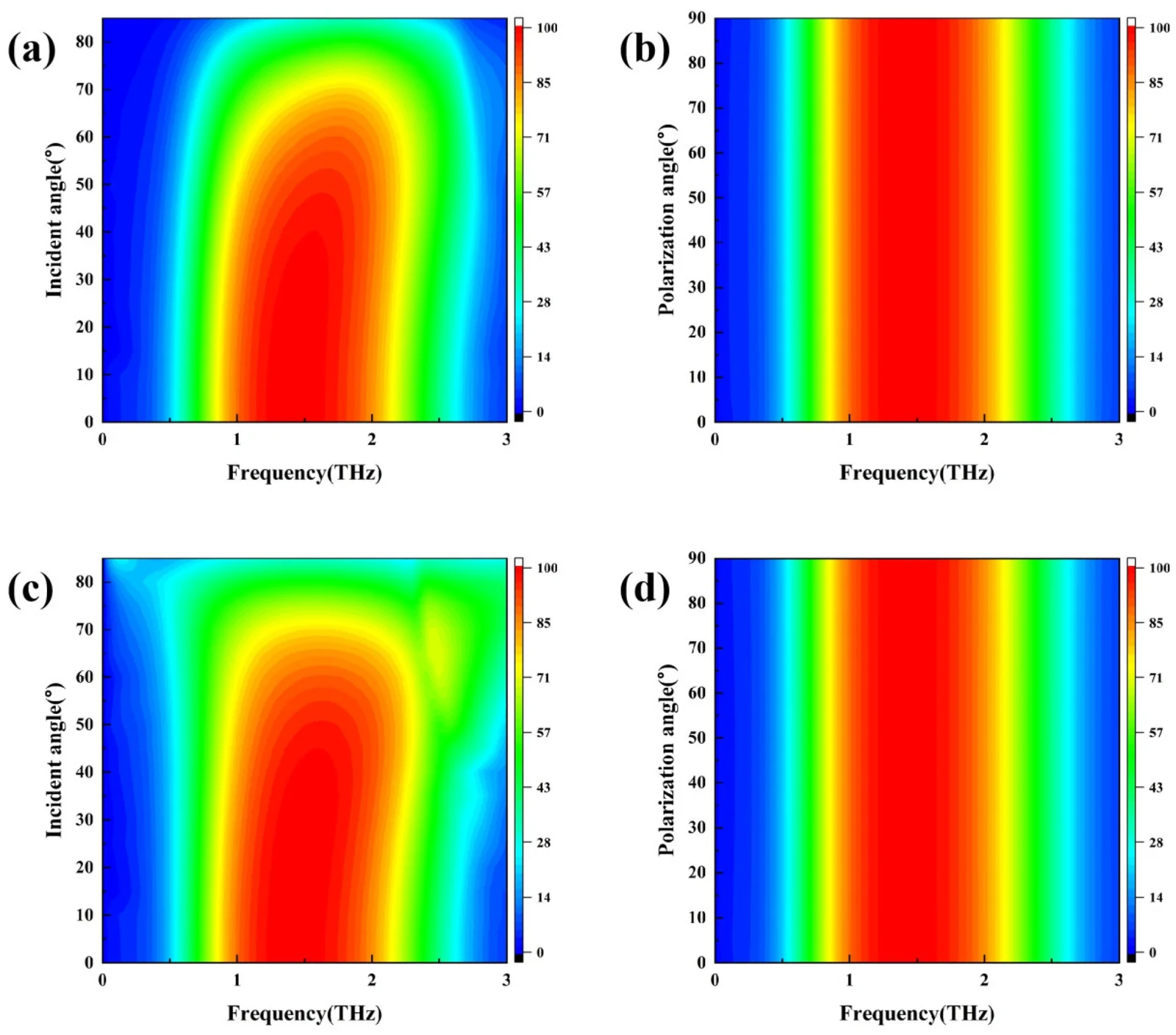
Contour maps of absorption under different incidence conditions: (**a**) *θ* and (**b**) ϕ at TE incidence and (**c**) *θ* and (**d**) ϕ at TM incidence.

To further quantify the sensitivity to incident direction, angular and polarization stability are examined in Figure 5. For TE incidence (Figure 5a), the absorber retains over 90% absorption in the 1.01–1.91 THz range for incident angles *θ* ≤ 55°, confirming excellent angular stability. Compared with previous studies [57], our absorber exhibits superior angular robustness. Beyond 55°, absorption performance gradually degrades. The high angular stability originates from the magnetic resonance mechanism supported by the Fabry-Pérot cavity formed between the top VO_2_ ring and the intermediate VO_2_ layer. For incident angles up to 55°, the normal component of the magnetic field, which primarily drives the resonance, remains sufficiently strong, maintaining impedance matching and high absorption. Beyond this threshold, the increasing tangential field component weakens the magnetic resonance and disrupts the cavity coupling, causing performance degradation. For varying *ϕ* from 0° to 90° in Figure 5b, polarization-angle dependence shows constant absorption in the 1.01–1.91 THz range, confirming polarization insensitivity arising from the square-ring symmetry. Such polarization insensitivity significantly enhances the practicality of the device by eliminating the need to consider the polarization direction. Figure 5c,d present the corresponding results under TM incidence, which are almost identical to those under TE incidence. The consistently identical responses for TE and TM incidences are attributed to the fourfold rotational symmetry of the square-ring structure, which ensures isotropic resonance excitation regardless of in-plane polarization. Thus, the absorber offers both robust angular stability and full polarization insensitivity, making it well suited for practical THz applications.

**Figure 6:**
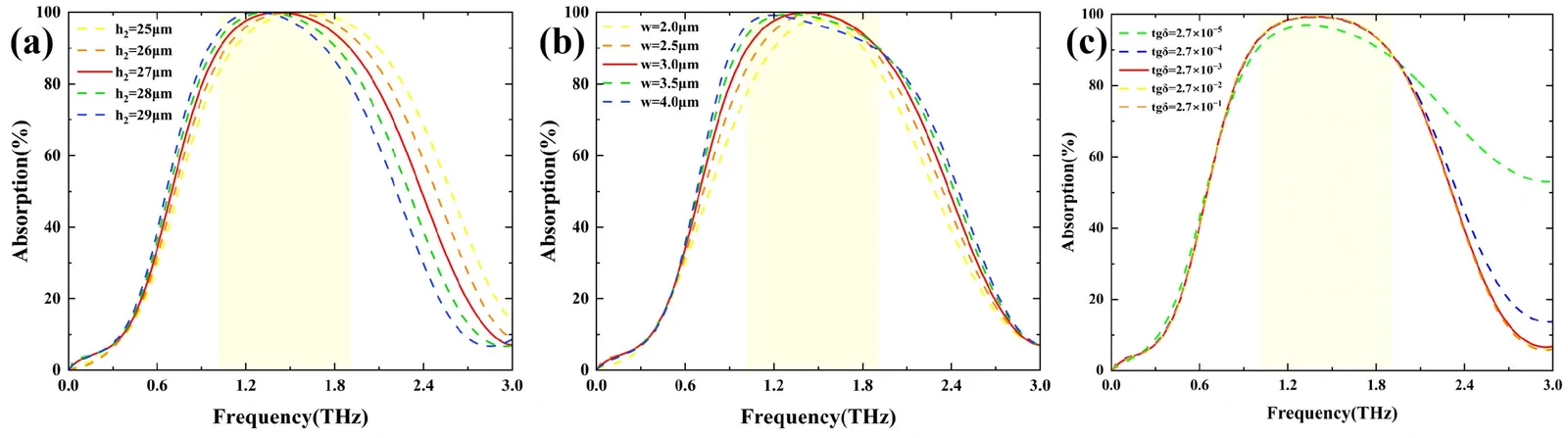
Absorption spectra of the absorber with different structural dimensions and material properties: (**a**) $h_{2}$, (**b**)  w, and (**c**) tgδ of the upper PI.

To further assess fabrication tolerance and optimize the absorption performance, we varied key structural parameters and material properties, as presented in Figure 6. As shown in Figure 6a, increasing the spacer thickness $h_{2}$ from 25 to 29 μm redshifts the absorption resonance and narrows the absorption bandwidth, consistent with the Fabry-Pérot resonator model [43,44]. As displayed in Figure 6b, widening the ring width *w* from 2 to 4 μm in steps of 0.5 μm similarly shifts the absorption peak to lower frequencies and reduces absorption bandwidth, which can be explained by the equivalent circuit model [58]: larger w increases both inductance L and capacitance C of the top ring, lowering the resonant frequency ($f\;\propto \;1/\sqrt{\mathrm{LC}\;}$). Despite these shifts, the absorber maintains highly efficient absorption under moderate dimensional deviations, indicating good fabrication robustness. As shown in Figure 6c, the absorption increases slightly when the loss tangent (tgδ) of the upper PI increases from 2.7 × $10^{-5}$ to 2.7 × $10^{-4}$. Further increasing tgδ, however, remains nearly unchanged absorption due to saturated dielectric loss. Hence, an appropriate loss tangent is essential for sustaining high-efficiency absorption.

### 3.2. Polarization Converter

To evaluate the PC type, several standard criteria are typically employed, as detailed below. For a normally incident TE wave polarized along the y-direction, the reflected field can be decomposed into co- and cross-polarized components [59,60,61,62]: ${\;E}_{r\;}\;=\;E_{\mathrm{xr}}e_{x}\;+\;E_{\mathrm{yr}}e_{y}\;=\;r_{\mathrm{ME}}\exp\left(j\varphi_{\mathrm{xy}}\right)E_{\mathrm{yi}}e_{x}\;+\;r_{\mathrm{EE}}\exp\left(j\varphi_{\mathrm{yy}}\right)E_{\mathrm{yi}}e_{y}$, where $E_{\mathrm{yi}}$ is the incident amplitude, $r_{\mathrm{ME}}$ and $\varphi_{\mathrm{xy}}$ are the amplitude and phase of the cross-polarization reflection coefficient, and $r_{\mathrm{EE}}$ and $\varphi_{\mathrm{yy}}$ correspond to those of the co-polarization component. Generally, an ideal linear-to-circular PC requires equal amplitudes ($r_{\mathrm{ME}}\;=\;r_{\mathrm{EE}}$) and a phase difference $\Delta \varphi =\;\varphi_{\mathrm{xy}}-\varphi_{\mathrm{yy}}\;=\;\pm 90{}^{\circ}$. Additionally, ellipticity χ and the axis ratio (AR) are given by:

(1) $$\left\{\begin{array}{c} \;\chi \;\;=\;\frac{2\left|r_{\mathrm{ME}}\right|\cdot \left|r_{\mathrm{EE}}\right|\sin\left(\Delta \varphi \right)}{{\left|r_{\mathrm{ME}}\right|}^{2}\;+\;{\left|r_{\mathrm{EE}}\right|}^{2}}\\ AR\;=\;10\log(\tan(\frac{1}{2}\;\times \;\arcsin(\frac{2\left|r_{\mathrm{ME}}\right|\;\cdot \;\left|r_{\mathrm{EE}}\right|\sin\left(∆\varphi \right)}{{\left|r_{\mathrm{ME}}\right|}^{2}\;+\;{\left|r_{\mathrm{EE}}\right|}^{2}}))) \end{array}\right.$$

**Figure 7:**
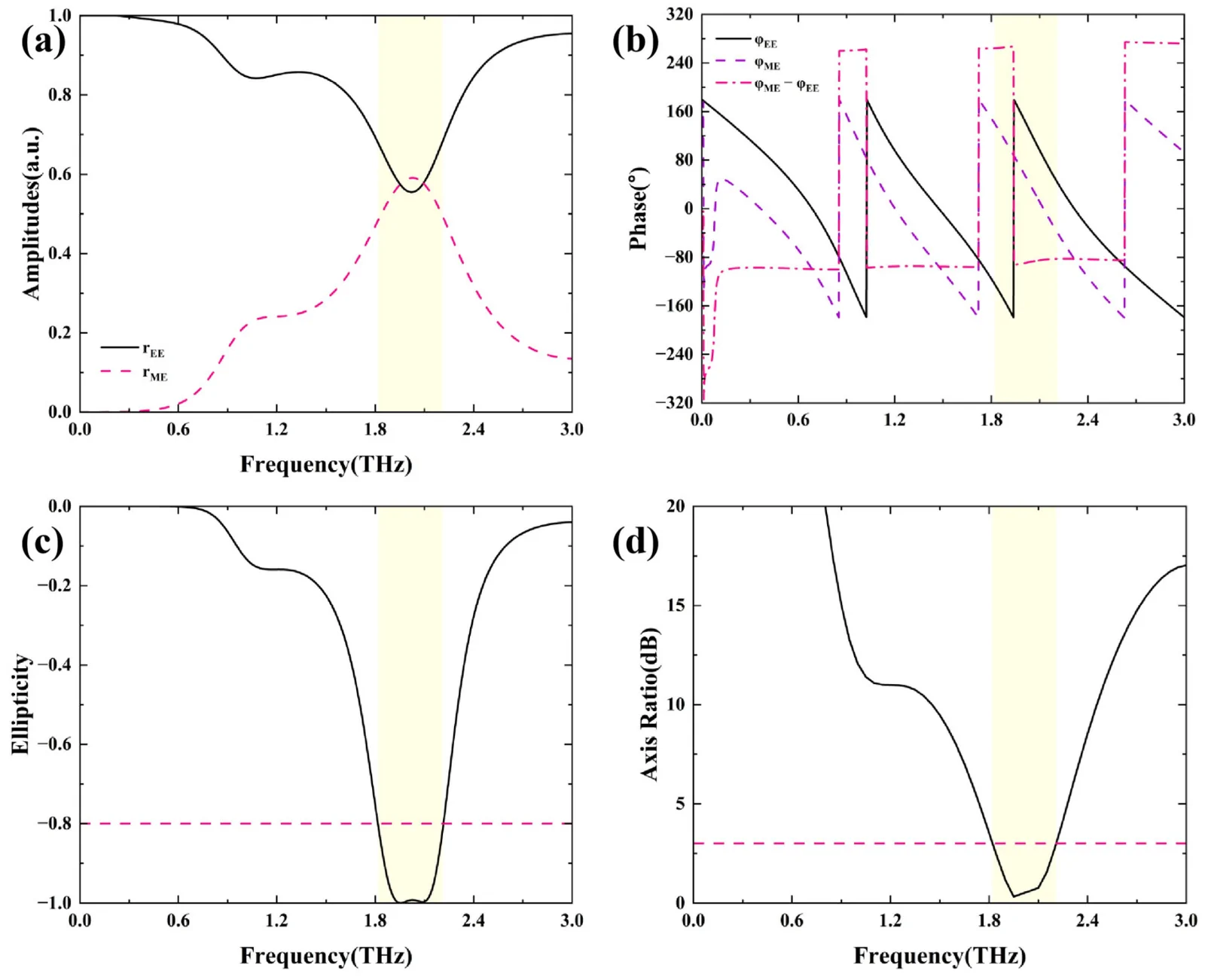
Different performances of the polarization converter: (**a**) reflection coefficient, (**b**) phase difference, (**c**) ellipticity, and (**d**) axial ratio.

Here, χ = ±1 corresponds to perfect CP, while χ = 0 indicates LP, and AR < 3 dB indicates good CP performance. To quantitatively assess our PC performance, Figure 7 shows the reflection intensity, phase difference, and calculated ellipticity and AR under normal TE incidence. In the 1.82–2.21 THz band, both $r_{\mathrm{ME}}$ and $r_{\mathrm{EE}}$ are nearly equal and close to unity (Figure 7a), and Δφ stays within −90° ± 5° (Figure 7b), enabling the LP-RHCP PC. Moreover, χ approaches −1 (Figure 7c), and AR remains below 3 dB (Figure 7d), enabling high-quality RHCP reflection. Therefore, the designed metasurface enables efficient linear-to-circular PC in the 1.82–2.21 THz band.

**Figure 8:**
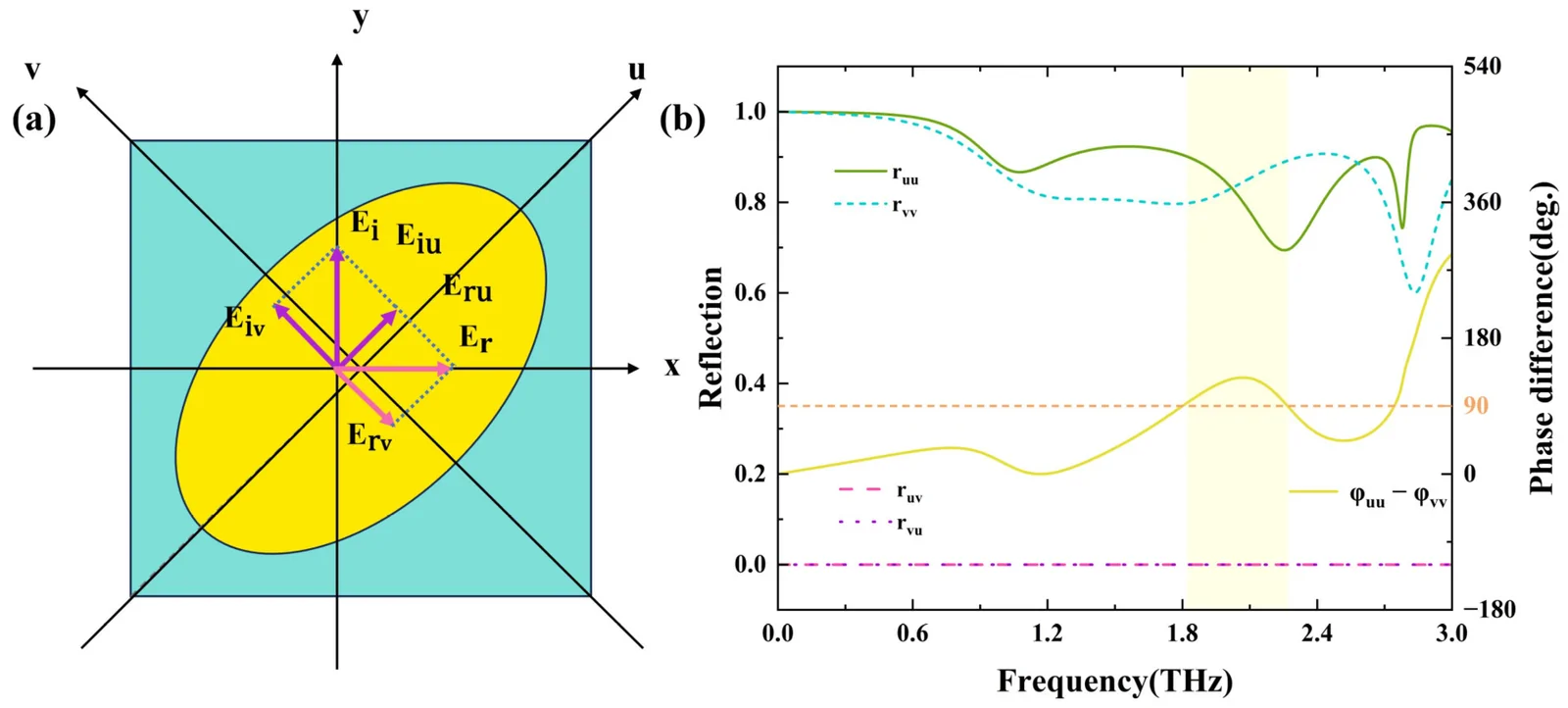
Physical mechanism of LCPC: (**a**) schematic of the *uv*-coordinate system, and (**b**) amplitude and phase difference.

To conveniently elucidate the physical mechanism underlying the PC, generally, a *uv*-coordinate system rotated by 45° relative to the original axes of the Cartesian coordinate is introduced [63]. This rotation aligns the frame with the symmetry axes of the elliptical patch, eliminating cross-polarization coupling. Consequently, the reflection coefficients along *u* and *v* directly represent the true eigenmodes, allowing the conditions for ideal circular polarization with equal amplitudes and a 90° phase difference to be evaluated without interference. The (*u*, *v*) decomposition thus provides a clear, physically intuitive interpretation of the conversion mechanism and is widely adopted in anisotropic metasurface analysis. As shown in Figure 8a, for a *y*-polarized incident wave, the field can be decomposed along the u- and *v*-axes by $E_{i\;}\;=\;\;\hat{y}E_{i}\;=\;\hat{u}E_{\mathrm{iu}}\;+\;\hat{v}E_{\mathrm{iv}}$, where $\hat{u}$ and $\hat{v}$ denote the unit vectors along the u- and v-directions, respectively, while $E_{\mathrm{iu}}$ and $E_{\mathrm{iv}}$ are the corresponding incident field orthogonal components [64]. Thus, the reflected wave can be decomposed in the uv-frame as: $E_{r}\;=\;\hat{u}E_{\mathrm{ru}}\;+\;\hat{v}E_{\mathrm{rv}\;\;}=\;\hat{u}\left(r_{\mathrm{uu}}E_{\mathrm{iu}}e^{i\varphi_{\mathrm{uu}}}\;+\;r_{\mathrm{uv}}E_{\mathrm{iv}}e^{i\varphi_{\mathrm{uv}}}\right)\;\;+\;\;\hat{v}\left(r_{\mathrm{vv}}E_{\mathrm{iv}}e^{i\varphi_{\mathrm{vv}}}\;+\;r_{\mathrm{vu}}E_{\mathrm{iv}}e^{i\varphi_{\mathrm{vu}}}\right)$, where, $r_{\mathrm{uu}}$ ($r_{\mathrm{uv}}$) and $\varphi_{\mathrm{uu}}$ ($\varphi_{\mathrm{uv}}$) represent the amplitude and phase of the co-polarized (cross-polarized) reflection component. Due to structural symmetry, cross-polarization coupling in the *uv* frame is negligible ($r_{\mathrm{uv}}\;=\;r_{\mathrm{vu}\;}=\;0$). When $r_{\mathrm{uu}\;}\;=\;r_{\mathrm{vv}}\;\approx \;1$ and $\Delta \varphi \;=\;\left|\varphi_{\mathrm{uu}\;}\;-\;{\;\varphi }_{\mathrm{vv}}\right|\;=\;90{}^{\circ}$, LCPC is achieved (Figure 8b).

**Figure 9:**
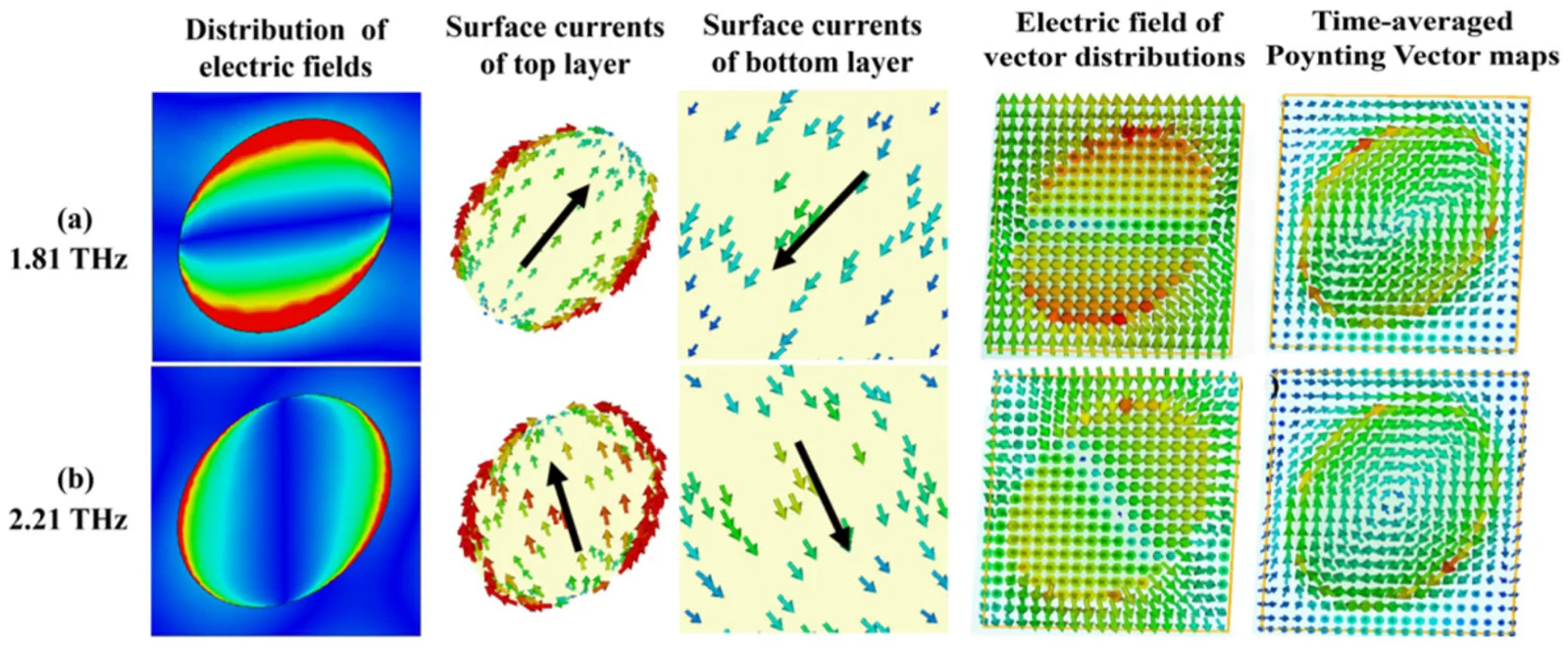
Field distributions on the top elliptical patch and bottom ground plane at two resonant frequencies: (**a**) 1.81 THz and (**b**) 2.21 THz.

To rigorously identify the resonance nature underpinning the LCPC, we examine the field distributions at the two resonant frequencies (1.81 and 2.21 THz), as presented in Figure 9. According to classical electromagnetic theory [65], antiparallel surface currents on the top and bottom metallic layers signify a magnetic dipole (MD) excitation (with the dipole moment normal to the current plane), whereas parallel currents indicate an electric dipole (ED) response. As shown in the first three columns of Figure 9, the currents on the elliptical patch and the bottom ground plane flow in opposite directions at both frequencies, confirming the MD-dominated response. This is further supported by the strong electric field localization at the patch edges, which is a hallmark of MD excitation arising from near-field capacitive coupling between two metallic layers. To further provide additional verification, we plot the electric field vector distributions and Poynting vector maps at two frequencies, as shown in the last two columns of Figure 9. At both frequencies, the electric field vectors exhibit circulating patterns around the patch centre, and the power flow streamlines form closed vortices, further confirming the characteristic signatures of MD resonances. In contrast, electric dipole excitations would manifest as parallel field orientations and outward radiating power flows. These vector-field observations, together with the antiparallel surface currents, conclusively demonstrate that the LCPC is governed by anisotropic MD resonances along the major and minor axes of the elliptical patch. This combined analysis not only resolves the resonance identification but also strengthens the physical interpretation of the conversion mechanism. Consequently, the PC is consistently attributed to orthogonal anisotropic MD eigenmodes. The required 90° phase difference originates from the anisotropic magnetic polarizabilities along the two axes, which yield distinct resonant frequencies and phase responses, while the equalized reflection amplitudes are achieved by optimizing the aspect ratio of the elliptical patch. Therefore, by tailoring the structural parameters, the reflection coefficients along these two orthogonal directions can be tuned to possess equal magnitude and a 90° phase difference over the desired band, ultimately enabling efficient LCPC [66].

**Figure 10:**
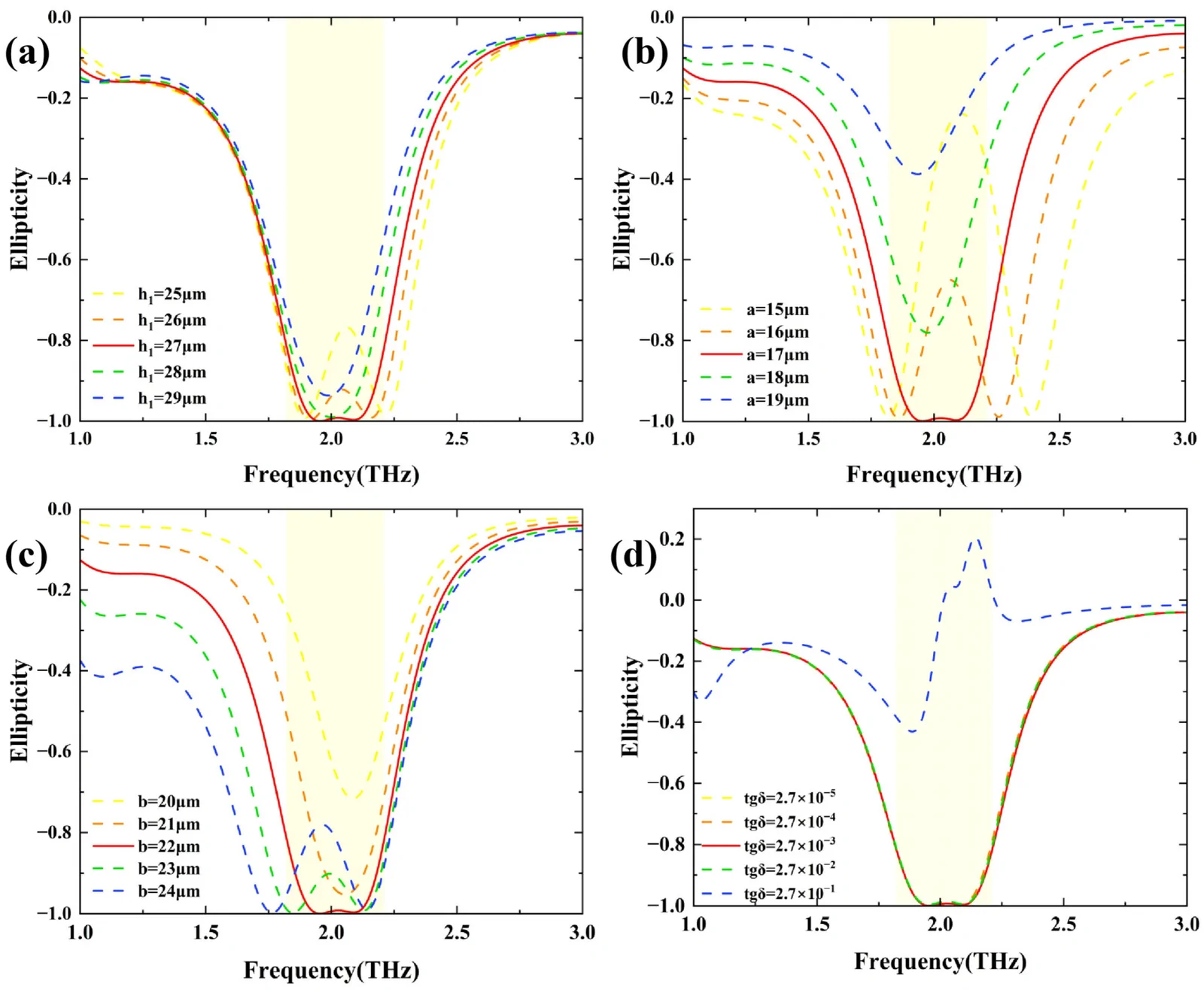
Ellipticity spectra of the polarization converter with different structural dimensions and material properties: (**a**) $h_{1}$, (**b**) a, (**c**) b, and (**d**) tgδ of the lower PI.

To further evaluate fabrication tolerance and material robustness, we analyze the influence of key geometric and material parameters on the ellipticity, as shown in Figure 10. Figure 10a presents the ellipticity spectra for varying $h_{1}$ from 25 to 29 μm while keeping other parameters unchanged. At $h_{1\;}=\;27\;\mu m,$ the ellipticity approaches −1 across the 1.82–2.21 THz band, indicating a desirable LCPC. When $h_{1}\;\leq \;27\;\mu m$, the dielectric layer is too thin to provide the required 90° phase delay, whereas for $h_{1}$ > 27 μm, the conversion performance degrades due to increased dielectric losses that disrupt impedance matching. Figure 10b shows the ellipticity spectra when the minor axis a is varied from 15 to 19 μm with the major axis fixed at b = 22 μm. At a = 15 μm, the ellipticity spectrum exhibits a dual-band behavior, indicating that the resonant frequencies along the two orthogonal directions are too widely separated. As a increases to 17 μm, the two bands merge into a single broadband, with ellipticity approaching −1 over the target range. Further increasing a to 19 μm makes the structure nearly circular, reducing anisotropy and causing ellipticity to approach zero, thus compromising the PC performance. Similarly, Figure 10c displays the ellipticity spectra for the major axis *b* swept from 20 to 24 μm with a fixed minor axis *a* = 17 μm. At b = 20 μm, the aspect ratio is close to unity, resulting in weak anisotropy and an near-zero ellipticity. At b = 22 μm, the aspect ratio (a : b ≈ 1.29) provides optimal anisotropy, yielding an ellipticity close to −1 across the target band. When b = 24 μm, the overly elongated major axis shifts the resonant frequency along the x-direction, degrading the performance. Based on these analyses, therefore, the optimal dimensions are determined as $h_{1}\;=\;27\;\mu m$, a = 17 μm, and b = 22 μm. Moreover, the ellipticity remains close to −1 within the target frequency range under moderate dimensional deviations, confirming good robustness against fabrication imperfections. Finally, Figure 10d presents the ellipticity spectra for various tgδ of the lower PI spacer. As observed, the ellipticity exhibits negligible variation and remains close to −1 as tgδ increases from 2.7 × 10^−5^ to 2.7 × 10^−2^, but the LCPC completely vanishes when tgδ reaches 2.7 × 10^−1^. Therefore, an appropriate loss tangent is important for sustaining high-efficiency LCPC.

**Figure 11:**
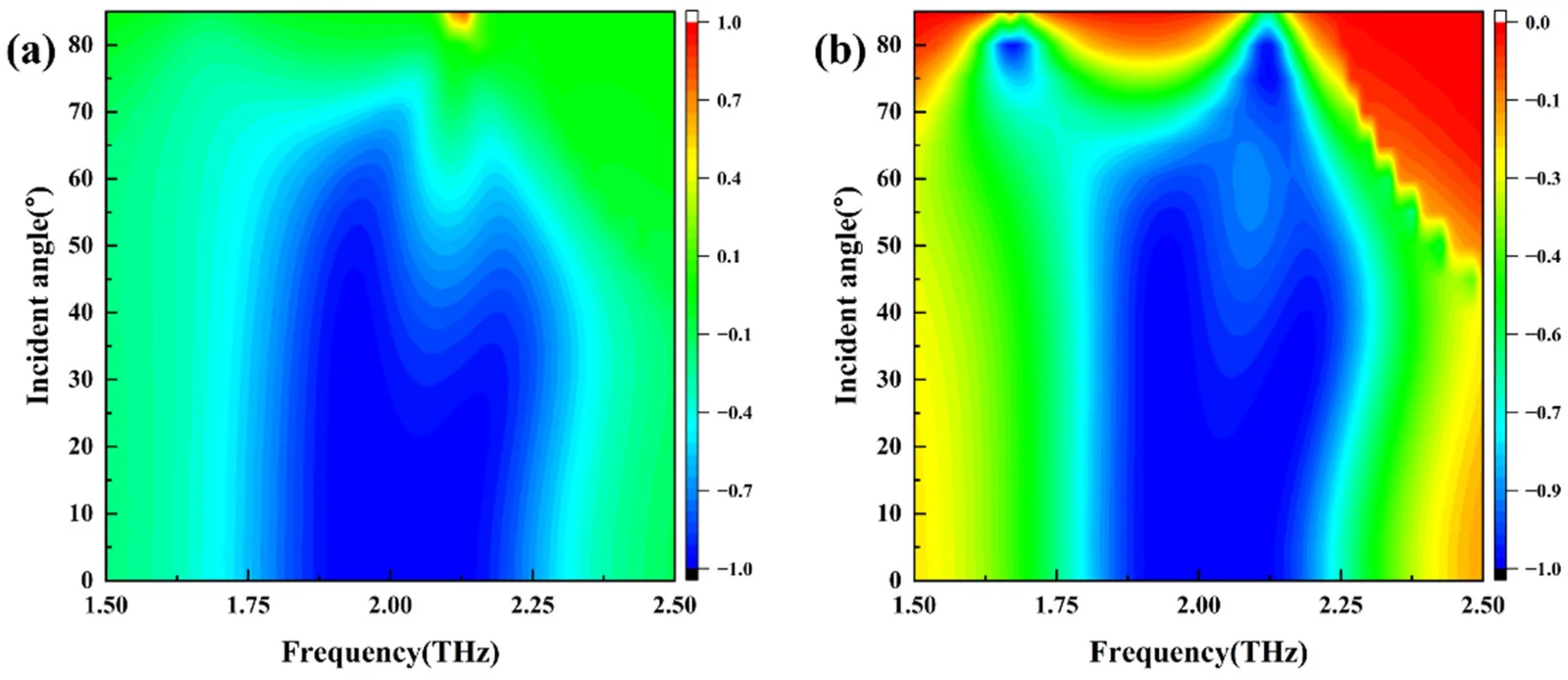
Dependence of ellipticity on the incidence angle *θ* under various polarization incidences: (**a**) TE mode and (**b**) TM mode.

To further assess the angular stability of the PC, we investigate the dependence of the ellipticity χ on incident angle *θ* for TE and TM polarizations, as shown in Figure 11. For TE incidence (Figure 11a), χ remains close to −1 across the 1.82–2.21 THz band for θ ≤ 35°, indicating stable conversion and high-quality RHCP output. Beyond 35°, χ gradually deviates from −1, reflecting degraded performance due to weakened resonance and phase-difference detuning from 90°.

For TM incidence, a similar trend is observed, as shown in Figure 11b. For θ ≤ 35°, χ stays near −1 over the 1.82–2.21 THz band. When θ > 35°, the ellipticity declines toward zero, indicating a transition toward linear polarization. These results confirm that the converter maintains robust performance up to 35° for both polarizations, beyond which the ellipticity deteriorates.

### 3.3. Performance Comparisons

To further highlight the novelty and advantages of the proposed design, a concise comparison is summarized in Table 1. Although previous VO_2_-based metasurfaces have been exploited to achieve continuous tunable absorption [21], switchable absorption and PC [27,51,62], and related works [26], these designs generally suffer from narrow bandwidth, limited angular stability, or polarization-sensitive responses, lacking comprehensive quantitative benchmarking. In contrast, our work delivers not only functional switching but also substantial performance gains: an absorption bandwidth of 0.90 THz, angular stability of up to 55°, and a higher-frequency LCPC band.

Critically, full polarization insensitivity in the absorbing state was rarely attained in previous switchable VO_2_ metasurfaces. Therefore, these combined advantages clearly demonstrate the superiority and versatility of our design, effectively overcoming the key limitations of prior works and establishing it as a compelling platform for reconfigurable, broadband, and polarization-robust THz devices.

## 4. Potential Fabrication Process and Measurement

**Figure 12:**
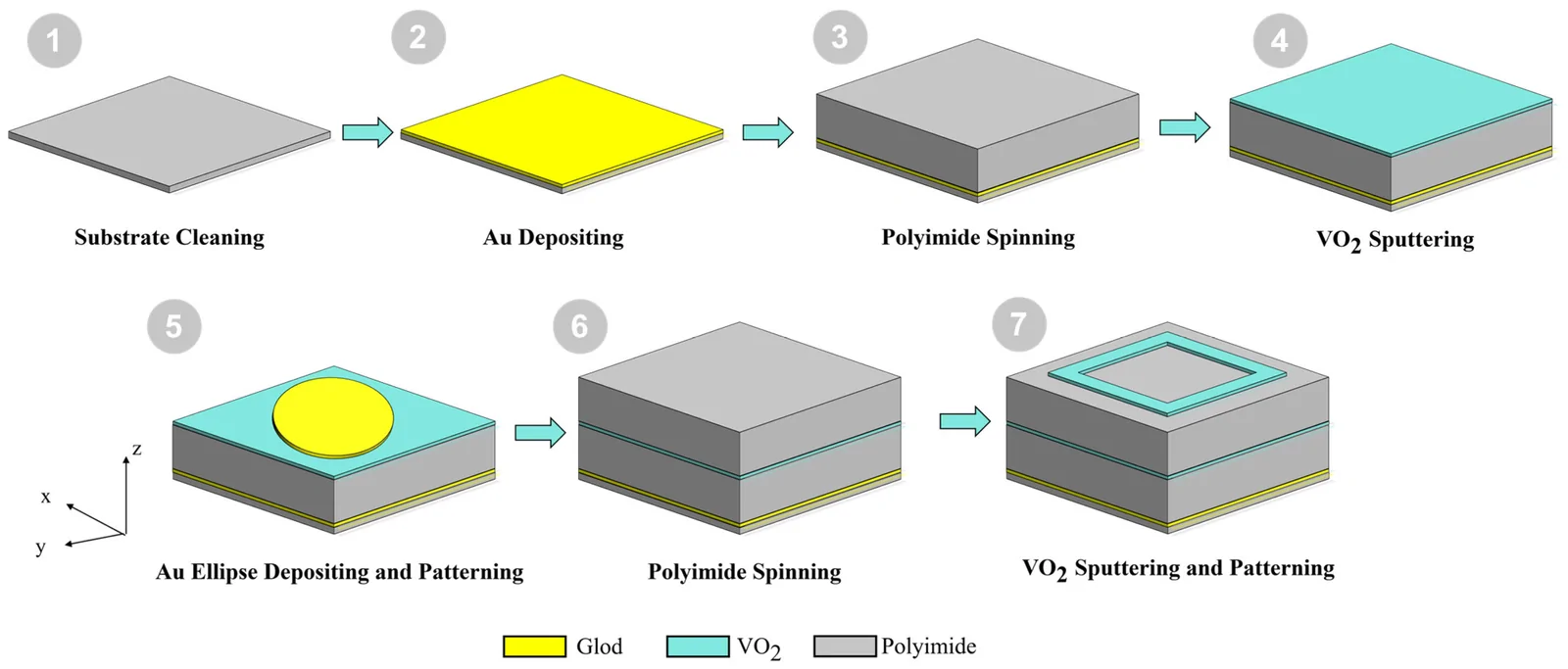
Fabrication process of the proposed metasurface.

Although experimental validation lies beyond the scope of this numerical study, we briefly outline the prospective fabrication process and measurement setup for the proposed metasurface. The six-layer structure can be fabricated using standard thin-film deposition and lithography techniques, as illustrated in Figure 12. The process begins with sequential cleaning of a quartz substrate using acetone, ethanol, and deionized water. Then, a bottom gold ground plane is deposited onto the substrate via electron-beam evaporation, followed by spin-coating and curing of the first PI layer to serve as the lower dielectric spacer. Subsequently, a VO_2_ film is deposited via magnetron sputtering and annealed to achieve the desired crystalline phase. An additional gold layer is deposited, patterned by photolithography, and etched to form the elliptical patch. Next, a second PI dielectric layer is spin-coated onto the patterned structure, followed by deposition of another VO_2_ thin film on the PI layer. Finally, the top VO_2_ film is patterned and selectively etched into the top square-ring structure using photolithography. Although the layer count exceeds that of single-function designs, the fabrication process relies on conventional thin-film deposition, photolithography, and lift-off processes, all of which are well established in metasurface manufacturing. Additionally, the VO_2_ phase transition near 68 °C can be thermally activated using a temperature-controlled heating stage integrated with the sample holder, enabling in situ switching between the insulating and metallic states. For THz characterization, a standard terahertz time-domain spectroscopy (THz-TDS) system in reflection geometry can be employed, with linearly polarized THz pulses generated by a photoconductive antenna. The co- and cross-polarized reflection coefficients are extracted from the measured time-domain waveforms, from which the absorption and PC performance can be derived. This fabrication and measurement protocol is well established for VO_2_-based THz metasurfaces and is directly transferable to our design.

## 5. Conclusions

In summary, we have accomplished the design and numerical verification of a reconfigurable multifunctional THz metasurface. The unit cell adopts a six-layer stacked configuration, with the sequential structure comprising a top VO_2_ ring, a first dielectric layer, an elliptical metal patch, an intermediate VO_2_ layer, a second dielectric spacer, and a bottom metal ground plane. By harnessing the insulator-to-metal transition of VO_2_, our structure enables switchable absorption and PC. Specifically, in the metallic state, it functions as a high-efficiency absorber, providing an average absorption exceeding 90% within the 1.01–1.91 THz range and exhibiting excellent angular stability up to 55°. In contrast, in the insulating state, it serves as an LCPC, achieving an ellipticity of nearly −1 and an axial ratio below 3 dB across the 1.82–2.21 THz band, with stable performance for incident angles up to 35°. The underlying physical mechanisms, including impedance matching, magnetic resonance excitation, and geometric anisotropy, have been systematically elucidated through simulations of electric field distributions, surface currents, and uv-coordinate decomposition. Overall, our VO_2_-integrated reconfigurable metasurface offers a feasible route for developing compact, tunable THz devices with switchable functions, showing significant application potential in wireless communication, intelligent sensing, and advanced imaging.

## Figures and Tables

**Table 1:** Performance comparison of the reported reconfigurable THz metasurfaces.

Ref	Functionality	Operation Mode	Average Absorption (%)	Absorption Range (THz)	Angular Stability of Absorption	LPC Range (THz)	Angular Stability of LPC
[21]	ABS	Continuous tuning	ABS > 90	1.27~2.16	≤60°	\	\
[26]	ABS Focusing	Functional switching	ABS = 97.5% ABS = 99.4%	0.42 0.93	\	\	\
[27]	ABS AT	Functional switching	ABS > 90	\	\	\	\
[51]	ABS LCPC	Functional switching	ABS > 90	0.67~0.95	≤33°	0.69~1.38	≤40°
[62]	ABS LCPC	Functional switching	ABS > 90	0.75~1.73	≤45°	0.96~1.47	\
This work	ABS LCPC	Functional switching	ABS > 90	1.01~1.91	≤55°	1.82~2.21	≤35°

## Data Availability

The original contributions presented in this study are included in the article. Further inquiries can be directed to the corresponding author.

## References

[1] Arbabi A., Arbabi E., Horie Y., Kamali S.M., Faraon A. (2017). Planar metasurface retroreflector. Nat. Photonics.

[2] Lin X., Gu W., Liu J., Zhang F. (2025). A near-infrared optical metasurfaces aggregate used for nondestructive testing of transparent targets. Opt. Laser Technol..

[3] Zheng W., Hao T., Li X., Luo W. (2024). Experimental validation of the horizontal resolution improvement by ultra-wideband metasurfaces for GPR systems. NDT E Int..

[4] John-Herpin A., Tittl A., Kühner L., Richter F., Huang S.H., Shvets G., Oh S.H., Altug H. (2023). Metasurface-enhanced infrared spectroscopy: An abundance of materials and functionalities. Adv. Mater..

[5] Khan S.A., Khan N.Z., Xie Y., Abbas M.T., Rauf M., Mehmood I., Runowski M., Agathopoulos S., Zhu J. (2022). Optical sensing by metamaterials and metasurfaces: From physics to biomolecule detection. Adv. Opt. Mater..

[6] Jahani Y., Arvelo E.R., Yesilkoy F., Koshelev K., Cianciaruso C., De Palma M., Kivshar Y., Altug H. (2021). Imaging-based spectrometer-less optofluidic biosensors based on dielectric metasurfaces for detecting extracellular vesicles. Nat. Commun..

[7] He S., Wang R., Luo H. (2022). Computing metasurfaces for all-optical image processing: A brief review. Nanophotonics.

[8] Kuznetsov A.I., Brongersma M.L., Yao J., Chen M.K., Levy U., Tsai D.P., Zheludev N.I., Faraon A., Arbabi A., Yu N. (2024). Roadmap for optical metasurfaces. ACS Photonics.

[9] Colburn S., Zhan A., Majumdar A. (2018). Metasurface optics for full-color computational imaging. Sci. Adv..

[10] Wang J., Li Y., Jiang Z.H., Shi T., Tang M.-C., Zhou Z., Chen Z.N., Qiu C.-W. (2020). Metantenna: When metasurface meets antenna again. IEEE Trans. Antennas Propag..

[11] Li L., Zhao H., Liu C., Li L., Cui T.J. (2022). Intelligent metasurfaces: Control, communication and computing. Elight.

[12] Iqbal R., Qureshi U.U.R., Jie C., Rahman Z.U., Jafar N. (2024). Polarization and incident angle independent multifunctional and multiband tunable THz metasurface based on VO2. Nanomaterials.

[13] Kuang X., Xu X., Li Y., Chao R., Liu J., Du Y. (2025). Broadband high-absorption tunable terahertz metamaterial absorber integrating gallium arsenide and vanadium dioxide. Opt. Mater. Express.

[14] Liu W., Tang C., Zheng R., Yi Y. (2025). Metamaterial terahertz absorbing device based on a hybrid structure of Dirac semimetal and graphene. Diam. Relat. Mater..

[15] Fallahi A., Perruisseau-Carrier J. (2012). Design of tunable biperiodic graphene metasurfaces. Phys. Rev. B Condens. Matter Mater. Phys..

[16] You J.W., Lan Z., Panoiu N.C. (2020). Four-wave mixing of topological edge plasmons in graphene metasurfaces. Sci. Adv..

[17] Jia W., Sensale-Rodriguez B. (2022). Terahertz metamaterial modulators based on wide-bandgap semiconductor lateral Schottky diodes. Opt. Mater. Express.

[18] Hao Y., Niu Z., Yang J., Wang M., Liu H., Qin Y., Su W., Zhang H., Zhang C., Li X. (2024). Self-powered terahertz modulators based on metamaterials, liquid crystals, and triboelectric nanogenerators. ACS Appl. Mater. Interfaces.

[19] Zhang H., Peng K., Jiang H., Li W., Zhao W. (2022). Multifunctional metasurfaces for switchable polarization selectivity and absorption. Opt. Express.

[20] Song Z., Chen A., Zhang J. (2020). Terahertz switching between broadband absorption and narrowband absorption. Opt. Express.

[21] Cheng Y.-Y., Meng D., Xu M.-Y., Liu Y., Zhuang P.-P., Lin D., Liu J., Chen Y.-S. (2023). Wide-band and narrow-band switchable terahertz absorber based on graphene. Results Phys..

[22] Zhang S., Chen K., Sun T., Song Q., Yi Z., Yi Y. (2025). Highly sensitive and tunable graphene metamaterial perfect absorber in the near-terahertz band. Coatings.

[23] Gupta S., Gosi V.C., Pareek P., Upender P. (2026). Dual-band graphene-assisted metamaterial absorber with machine learning integration for high-sensitivity THz biosensing. Sci. Rep..

[24] Asgari S., Fabritius T. (2025). Multi-band terahertz graphene-based anisotropic metamaterial absorber comprised of two circular split ring resonator arrays with two gaps and a connection rod. Opt. Quantum Electron..

[25] Hou Y., Zhang C., Wang C. (2020). High-efficiency and tunable terahertz linear-to-circular polarization converters based on all-dielectric metasurfaces. IEEE Access.

[26] Niu J., Gong H., Xu C., Li C., Hui Q., Tian R., Mo W. (2024). Switchable multifunctional metasurface based on vanadium dioxide absorbing and focusing at the same frequency. Appl. Opt..

[27] Ren Y., Zhou T., Jiang C., Tang B. (2021). Thermally switching between perfect absorber and asymmetric transmission in vanadium dioxide-assisted metamaterials. Opt. Express.

[28] Xiao B., Wang X., Gao M. (2025). Research on VO2-based absorbing/polarization conversion terahertz devices. Opt. Commun..

[29] Gao J., Deng X.-H., Geng J., Xiong Z., Song B. (2024). A Vanadium Dioxide-Based THz Metasurface for Dual-Function Switching Between Wideband Absorption and Polarization Conversion. J. Electron. Mater..

[30] Yan D., Meng M., Li J., Li J., Li X. (2020). Vanadium dioxide-assisted broadband absorption and linear-to-circular polarization conversion based on a single metasurface design for the terahertz wave. Opt. Express.

[31] Song Z., Zhang J. (2020). Achieving broadband absorption and polarization conversion with a vanadium dioxide metasurface in the same terahertz frequencies. Opt. Express.

[32] Li J.-S., Li X.-J. (2022). Switchable tri-function terahertz metasurface based on polarization vanadium dioxide and photosensitive silicon. Opt. Express.

[33] Li J.-D., Lv Y., Wang Y.-H., Li X.-M., Pan Y., Cao H.-Z., Ma R.-D., Xu S.-T. (2025). VO_2_-integrated multifunctional terahertz metasurface: From polarization converter to absorber. Phys. Lett. A.

[34] Jia Y., Wang G., Zhang X., Li M., Miao F., Gao Y. (2023). Terahertz multi-band absorber and dual-bandwidth polarization converter based on VO2 and graphene. Results Phys..

[35] Chen Z., Chen J., Tang H., Shen T., Zhang H. (2023). Multifunctional terahertz device based on a VO_2_-assisted metamaterial for broadband absorption, polarization conversion, and filtering. Opt. Mater. Express.

[36] Wang X., He X., Tang C., Shui B., Yi Z. (2025). A terahertz band multifunctional metamaterial transmission–absorption switching device based on vanadium dioxide. Dalton Trans..

[37] Nie R., He C., Zhang R., Song Z. (2023). Vanadium dioxide-based terahertz metasurfaces for manipulating wavefronts with switchable polarization. Opt. Laser Technol..

[38] Wang T., Zhang Y., Zhang H., Cao M. (2020). Dual-controlled switchable broadband terahertz absorber based on a graphene-vanadium dioxide metamaterial. Opt. Mater. Express.

[39] Huang J., Li J., Yang Y., Li J., Li J., Zhang Y., Yao J. (2020). Active controllable dual broadband terahertz absorber based on hybrid metamaterials with vanadium dioxide. Opt. Express.

[40] Serebryannikov A.E., Lakhtakia A., Ozbay E. (2022). Thermally switchable, bifunctional, scalable, mid-infrared metasurfaces with VO2 grids capable of versatile polarization manipulation and asymmetric transmission. Opt. Mater. Express.

[41] Huang J., Li J., Yang Y., Li J., Li J., Zhang Y., Yao J. (2020). Broadband terahertz absorber with a flexible, reconfigurable performance based on hybrid-patterned vanadium dioxide metasurfaces. Opt. Express.

[42] Song Z., Wei M., Wang Z., Cai G., Liu Y., Zhou Y. (2019). Terahertz absorber with reconfigurable bandwidth based on isotropic vanadium dioxide metasurfaces. IEEE Photonics J..

[43] Wu G., Jiao X., Wang Y., Zhao Z., Wang Y., Liu J. (2021). Ultra-wideband tunable metamaterial perfect absorber based on vanadium dioxide. Opt. Express.

[44] Born N., Crunteanu A., Humbert G., Bessaudou A., Koch M., Fischer B.M. (2015). Switchable THz filter based on a vanadium dioxide layer inside a Fabry–Pérot cavity. IEEE Trans. Terahertz Sci. Technol..

[45] Wang Y., Chen Y., Liu F., Chen L., Ji K., Wang X., Ji X. (2025). Vanadium dioxide enabled polarization insensitive tunable broadband terahertz metamaterial absorber. Sci. Rep..

[46] Li X., Tang S., Ding F., Zhong S., Yang Y., Jiang T., Zhou J. (2019). Switchable multifunctional terahertz metasurfaces employing vanadium dioxide. Sci. Rep..

[47] Wang C.-L., Tian Z., Xing Q.-R., Gu J.-Q., Liu F., Hu M.-L., Chai L., Wang Q.-Y. (2010). Photo-induced insulator-metal transition of silicon-based VO 2 nanofilm by THz time domain spectroscopy. Wuli Xuebao.

[48] Gavdush A.A., Zhelnov V.A., Dolganov K.B., Bogutskii A.A., Garnov S.V., Burdanova M.G., Ponomarev D.S., Shi Q., Zaytsev K.I., Komandin G.A. (2025). Insulator–metal transition in VO2 film on sapphire studied by broadband dielectric spectroscopy. Sci. Rep..

[49] Zhuang L., Zhang W., Chao M., Liu Q., Cheng B., Song G., Liu J. (2024). Terahertz broadband tunable multifunctional metasurface based on VO2. Opt. Mater. Express.

[50] Mou N., Tang B., Li J., Dong H., Zhang L. (2022). Switchable ultra-broadband terahertz wave absorption with VO2-based metasurface. Sci. Rep..

[51] Li Y., Zeng L., Zhang H., Zhang D., Xia K., Zhang L. (2022). Multifunctional and tunable metastructure based on VO2 for polarization conversion and absorption. Opt. Express.

[52] Ding F., Zhong S., Bozhevolnyi S.I. (2018). Vanadium dioxide integrated metasurfaces with switchable functionalities at terahertz frequencies. Adv. Opt. Mater..

[53] Barzegar-Parizi S., Khavasi A. (2019). Designing dual-band absorbers by graphene/metallic metasurfaces. IEEE J. Quantum Electron..

[54] Morozov M.Y., Popov V.V. (2025). Total resonant terahertz absorption enabled by large-scale adjustable admittance inverse matching in a metal groove with graphene. J. Phys. D Appl. Phys..

[55] Qu W., Li Z., Li G., Deng H., Xiong Z. (2024). High-performance terahertz microfluidic sensors based on Fabry–Perot resonance. Opt. Mater..

[56] Zhang Y., Sun X., Liu L., Qin G., Yu H., Li Z. (2024). A perfect absorber based on a VO2-tunable Fabry–Perot cavity: An analysis of periodic oscillation absorption characteristics. Phys. Scr..

[57] Liao S., Sui J., Zhang H. (2022). Switchable ultra-broadband absorption and polarization conversion metastructure controlled by light. Opt. Express.

[58] Bilal R., Baqir M., Choudhury P., Naveed M., Ali M., Rahim A. (2020). Ultrathin broadband metasurface-based absorber comprised of tungsten nanowires. Results Phys..

[59] Lin B., Lv L., Guo J., Liu Z., Ji X., Wu J. (2020). An ultra-wideband reflective linear-to-circular polarization converter based on anisotropic metasurface. IEEE Access.

[60] Gao X., Li K., Wu X., Xue C., Wang G., Xie X., Qin M. (2022). Ultra-wideband linear-to-circular polarizer realized by bi-layer metasurfaces. Opt. Express.

[61] Barkabian M., Dalvand N., Zandi H., Granpayeh N. (2023). Terahertz linear to circular polarization converter based on reflective metasurface. Sci. Iran..

[62] Jiang Y., Zhao H., Wang L., Wang J., Cao W., Wang Y. (2019). Broadband linear-to-circular polarization converter based on phosphorene metamaterial. Opt. Mater. Express.

[63] Gao X., Han X., Cao W.-P., Li H.O., Ma H.F., Cui T.J. (2015). Ultrawideband and high-efficiency linear polarization converter based on double V-shaped metasurface. IEEE Trans. Antennas Propag..

[64] Cildir A., Tahir F.A., Farooq M., Zahid A., Imran M., Abbasi Q.H. (2025). A highly efficient and broadband metasurface for linear-to-linear and linear-to-circular polarization conversion in reflection mode. Photonics Nanostruct.-Fundam. Appl..

[65] Khan M.I., Khalid Z., Tahir F.A. (2019). Linear and circular-polarization conversion in X-band using anisotropic metasurface. Sci. Rep..

[66] Zhang X., Ye H., Zhao Y., Zhang H. (2022). Linear-to-circular polarization converter with adjustable bandwidth realized by the graphene transmissive metasurface. Plasmonics.

